# SCH23390 Reduces Methamphetamine Self-Administration and Prevents Methamphetamine-Induced Striatal LTD

**DOI:** 10.3390/ijms21186491

**Published:** 2020-09-05

**Authors:** Yosef Avchalumov, Wulfran Trenet, Juan Piña-Crespo, Chitra Mandyam

**Affiliations:** 1 VA San Diego Healthcare System, San Diego, CA 92161, USA; YAvchalumov@vapop.ucsd.edu (Y.A.); wtrenet@vapop.ucsd.edu (W.T.); 2 Department of Molecular Medicine, Scripps Research, La Jolla, CA 92037, USA; jcpina-crespo@scripps.edu; 3 Department of Anesthesiology, University of California San Diego, San Diego, CA 92161, USA

**Keywords:** SCH23390, self-administration, field excitatory postsynaptic potential (fEPSP), methamphetamine, dorsal striatum, postsynaptic density protein (PSD-95)

## Abstract

Extended-access methamphetamine self-administration results in unregulated intake of the drug; however, the role of dorsal striatal dopamine D_1_-like receptors (D_1_Rs) in the reinforcing properties of methamphetamine under extended-access conditions is unclear. Acute (ex vivo) and chronic (in vivo) methamphetamine exposure induces neuroplastic changes in the dorsal striatum, a critical region implicated in instrumental learning. For example, methamphetamine exposure alters high-frequency stimulation (HFS)-induced long-term depression in the dorsal striatum; however, the effect of methamphetamine on HFS-induced long-term potentiation (LTP) in the dorsal striatum is unknown. In the current study, dorsal striatal infusion of SCH23390, a D_1_R antagonist, prior to extended-access methamphetamine self-administration reduced methamphetamine addiction-like behavior. Reduced behavior was associated with reduced expression of PSD-95 in the dorsal striatum. Electrophysiological findings demonstrate that superfusion of methamphetamine reduced basal synaptic transmission and HFS-induced LTP in dorsal striatal slices, and SCH23390 prevented this effect. These results suggest that alterations in synaptic transmission and synaptic plasticity induced by acute methamphetamine via D_1_Rs could assist with methamphetamine-induced modification of corticostriatal circuits underlying the learning of goal-directed instrumental actions and formation of habits, mediating escalation of methamphetamine self-administration and methamphetamine addiction-like behavior.

## 1. Introduction

Methamphetamine is abused worldwide for its stimulant-like effects, and methamphetamine use disorder (MUD) is a public health issue in the United States [1,2]. The adverse effects of MUD are both short- and long-term, including cardiac arrhythmias, insomnia, confusion, cognitive impairments, and addiction, and MUD-associated neurotoxicity produces long-term changes in brain structure and function [3,4,5]. The exact mechanisms that underlie the addiction-related responses are not completely understood; however, they are thought to be caused by functional and structural changes in the dopaminergic system in the striatum [1,6,7]. Methamphetamine is a substrate for the dopamine transporter and greatly increases the concentration of synaptic dopamine, serotonin, and noradrenaline by redistributing these neurotransmitters from synaptic vesicles to the cytosol [8]. Methamphetamine also affects glutamate levels in the brain, which, in turn, can cause long-lasting changes in neurotransmission in the brain, particularly the striatum [9,10,11]. These effects of methamphetamine could be occurring in the dorsal striatum to enhance the brain’s reactivity to drug context, drug cues, and the drug itself [12].

Dopamine affects many physiological functions, such as the control of coordinated movements, as well as motivated and emotional behaviors by altering the neuroplasticity in the striatum [13,14]. Therefore, methamphetamine-induced alterations in the function of the nigrostriatal circuit in the dorsal striatum are hypothesized to underlie MUD. For example, the dorsal striatum does not play a critical role in initial drug reinforcement; however, it is hypothesized to play a role in the acquisition and maintenance of drug self-administration, as well as the eventual habitual behavior that develops after overtraining [15,16]. More notably, the dorsal striatum regulates the transition from controlled to uncontrolled drug intake which is characterized by escalation in rodents and binging in human subjects [17], and which could be due to the function of the dorsal striatum in stimulus-response habits [16,18].

Loss-of-function studies of global dopamine D_1_-like receptor (D_1_R) knock-out in mice demonstrate that these mice do not self-administer cocaine compared with wild-type controls [19]. Notably, site-specific knockdown of D_1_Rs via RNA interference in the dorsal striatum increases methamphetamine self-administration, which may be indicative of an enhancement in the reinforcing properties of methamphetamine [20]. Site-specific knockdown of D_1_Rs in the dorsal striatum did not alter the response to sucrose, a natural reward. These studies indicate that global knockdown of D_1_Rs may have distinct effects on cocaine self-administration, and that in vivo dorsal striatal knockdown of D_1_Rs plays a role in enhancing MUD. Loss-of-function studies of global dopamine D_2_-like receptor (D_2_R) knock-out in mice show that, in these mice, methamphetamine does not produce neurotoxicity or cell death. It is important to note that, while D_1_Rs are mostly expressed on GABAergic medium spiny neurons (MSNs) in the dorsal striatum, D_2_Rs are expressed on presynaptic dopamine neurons projecting from the substantia nigra (autoreceptors) and on GABAergic MSNs (heteroreceptors) [21]. Furthermore, new evidence demonstrates that D_2_ heteroreceptors, especially in the dorsal striatum, play an important role in regulating dopamine release in response to psychostimulants [21], thus suggesting that D_2_Rs also play a role in enhancing MUD. Pharmacological studies indicate that methamphetamine acts as an indirect agonist at D_1_Rs and D_2_Rs [22]; however, it produces its rewarding effects through D_1_Rs. For example, systemic D_1_R antagonism by SCH23390, a potent D_1_R antagonist [23], reduces amphetamine-induced locomotor activity and locomotor sensitization, as well as multiple addiction-related behaviors including reward, self-administration and priming-induced drug seeking [24,25,26,27,28,29]. Systemic D_2_R antagonism does not affect methamphetamine reward and self-administration [24,28]. Microinjection of SCH23390 into the dorsal striatum prevents methamphetamine-induced loss of striatal dopamine transporters, and it reduces methamphetamine-induced hyperlocomotion and some aspects of amphetamine-induced timing impulsivity [30,31,32]. However, the specific role of D_1_Rs in the dorsal striatum in methamphetamine reinforcement is unknown, leading to its investigation in this study. Next, dendritic spine density in the ventral striatum is positively correlated with addiction-like behavior, suggesting that levels in postsynaptic density protein (PSD-95) could be predicted by enhanced reinforcing properties of drugs of abuse [33,34]. However, such alterations in the expression of PSD-95 in the dorsal striatum in animal models of methamphetamine addiction were minimally investigated, leading to their exploration in this study.

In the dorsal striatum, D_1_Rs and D_2_Rs play a role in mediating synaptic transmission and synaptic plasticity of GABAergic MSNs [33,35,36,37,38], such that activation of these receptors produces long-term potentiation (LTP) and long-term depression (LTD). In the context of methamphetamine exposure, protracted withdrawal from experimenter-delivered methamphetamine or self-administered methamphetamine prevents high-frequency stimulation (HFS)-induced LTD in the dorsal striatum and produces LTP in the dorsal striatum [39,40]. However, no studies evaluated the ongoing effect of acute methamphetamine on striatal plasticity, specifically, HFS-induced LTP in the dorsal striatum. We, therefore, investigated whether acute methamphetamine treatment alters HFS-induced LTP in the dorsal striatum, and if these effects occurred via activation of D_1_Rs.

## 2. Results

### 2.1. Escalation of Self-Administration Is Evident in Rats Taking Methamphetamine and Not Saline

**Figure 1:**
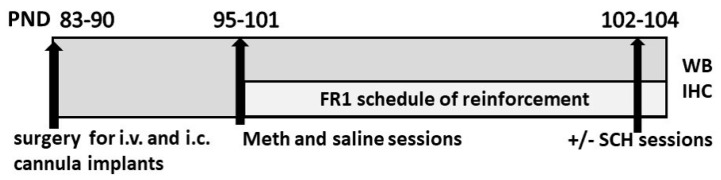
Schematic of experimental design indicating postnatal day of the rats, surgery for cannula implants, self-administration sessions, and euthanasia. Postnatal days (PND), intravenous (i.v.), intracranial (i.c.), methamphetamine (Meth), SCH23390 (SCH), Western blotting (WB), immunohistochemistry (IHC).

**Figure 2:**
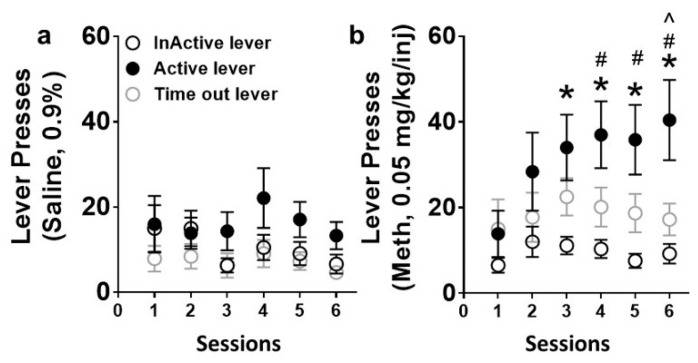
Self-administration data from saline (**a**) and methamphetamine (**b**) rats. (**a**) Active, inactive, and timeout lever responses during extended 6 h access sessions of saline self-administration. (**b**) Active, inactive, and timeout lever responses during extended 6 h access sessions of methamphetamine self-administration. *n* = 9 saline and *n* = 20 methamphetamine rats. Data are expressed as mean ± standard error of the mean (SEM). * *p* < 0.05 vs. inactive lever responses, ^ *p* < 0.05 vs. timeout lever responses, ^#^ *p* < 0.05 vs. session 1 by repeated-measures ANOVA.

Rats self-administered either saline or methamphetamine in an extended-access schedule of reinforcement (Figure 1). Lever presses on the active and inactive levers for saline rats and methamphetamine rats were analyzed separately to determine lever discrimination and escalation of self-administration. Lever responses during timeout were also analyzed, as responses during timeout could indicate the inability to suppress unrewarded behavior or compulsivity [41]. Repeated-measures two-way ANOVA did not reveal a lever × session interaction (F (10, 120) = 0.6, *p* = 0.7) or a main effect of lever (F (2, 24) = 2.7, *p* = 0.08) or session (F (5, 120) = 1.6, *p* = 0.1) in rats that self-administered saline (Figure 2a). Repeated-measures two-way ANOVA did not reveal a lever × session interaction (F (10, 285) = 1.0, *p* = 0.3); however, a main effect of lever (F (2, 57) = 9.8, *p* = 0.0002) and session (F (5, 285) = 2.2, *p* = 0.05) was identified in rats that self-administered methamphetamine (Figure 2b). Post hoc analysis demonstrated a higher number of active lever presses compared with inactive lever presses, as well as a higher number of active lever presses in sessions 3–6 compared to session 1. Post hoc analysis also revealed an increase in active lever responses compared with timeout lever responses in session 6.

### 2.2. Microinjection of SCH23390 into the Dorsal Striatum Reduces Self-Administration of Methamphetamine without Altering Self-Administration of Saline

**Figure 3:**
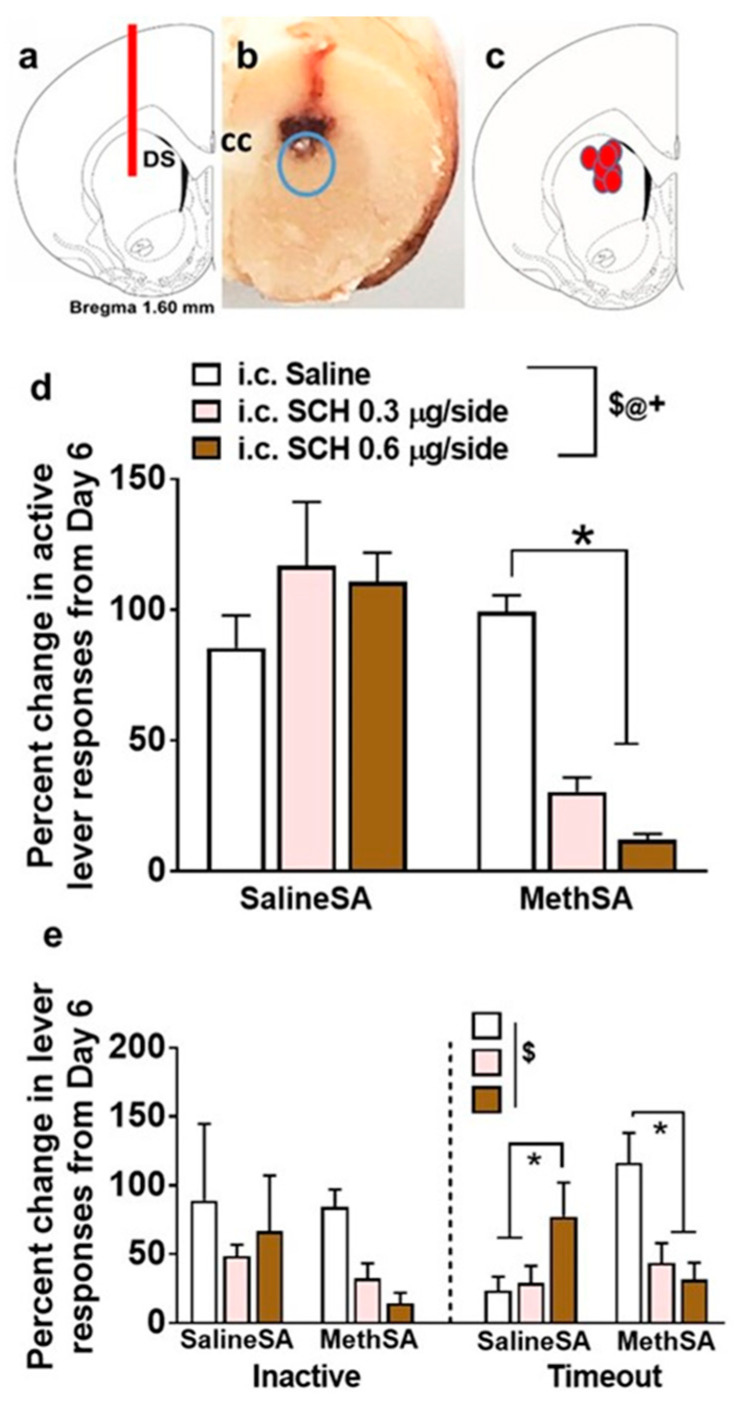
SCH23390 reduces methamphetamine self-administration. (**a**) Schematic of a coronal slice representing the area of the dorsomedial striatum with cannula placement for i.c. injections. (**b**) Photomicrograph of the rat brain indicating the area of cannula placement after removal of the i.c. cannula. The infusion site of SCH23390 is indicated in a blue circle. (**c**) Schematic of a coronal slice indicating the placements of cannula tip from each rat. (**d**) Change in active lever responses in saline and methamphetamine rats after infusion of vehicle (saline) or doses of SCH23390 expressed as a percentage change from session 6 of self-administration. (**e**) Change in inactive and timeout lever responses in saline and methamphetamine rats after infusion of vehicle (saline) or doses of SCH23390 expressed as a percentage change from session 6 of self-administration. Intracranial infusions were conducted as a within-subject design with saline infusions conducted first, followed by a 0.3 µg dose of SCH23390 and a 0.6 µg dose of SCH23390 infusions. *n* = 4 saline and *n* = 7 methamphetamine rats. Data are expressed as mean ± SEM. ^$^ *p* < 0.05, significant group × treatment interaction; ^@^ *p* < 0.05, significant main effect of methamphetamine; ^+^ *p* < 0.05, significant main effect of SCH23390 by repeated-measures ANOVA; * *p* < 0.05 vs. vehicle (saline) responses by post hoc analysis.

We next determined whether microinjection of SCH23390 altered behavior in rats self-administering saline and methamphetamine. When active lever responses were compared, repeated-measures two-way ANOVA revealed a significant group × SCH23390 interaction (F (2, 18) = 15.3, *p* = 0.0001), a main effect of group (F (1, 9) = 101.3, *p* < 0.0001), and a main effect of SCH23390 (F (2, 18) = 3.8, *p* = 0.03). Post hoc analysis showed a lower number of lever presses in methamphetamine rats after SCH23390 infusion compared with saline infusion (Figure 3d). When inactive lever responses were compared, repeated-measures two-way ANOVA did not detect a group × SCH23390 interaction (F (2, 18) = 0.6, *p* = 0.51), a main effect of group (F (1, 9) = 1.3, *p* = 0.28), or a main effect of SCH23390 (F (2, 18) = 3.1, *p* = 0.06; Figure 3e). When timeout lever responses were compared, repeated-measures two-way ANOVA revealed a significant group × SCH23390 interaction (F (2, 18) = 13.16, *p* = 0.0003), without a main effect of group (F (1, 9) = 1.0, *p* = 0.33) or main effect of SCH23390 (F (2, 18) = 3.0, *p* = 0.07). Post-hoc analysis showed higher number of lever presses in saline rats after a 0.6 µg dose of SCH23390 compared with saline and a 0.3 µg dose (Figure 3e). Post hoc analysis also showed lower number of lever presses in methamphetamine rats after 0.3 and 0.6 µg doses of SCH23390 compared with saline infusion (Figure 3e).

### 2.3. Methamphetamine Self-Administration Enhances Expression of PSD-95 in the Dorsal Striatum and SCH23390 Reduces This Effect

**Figure 4:**
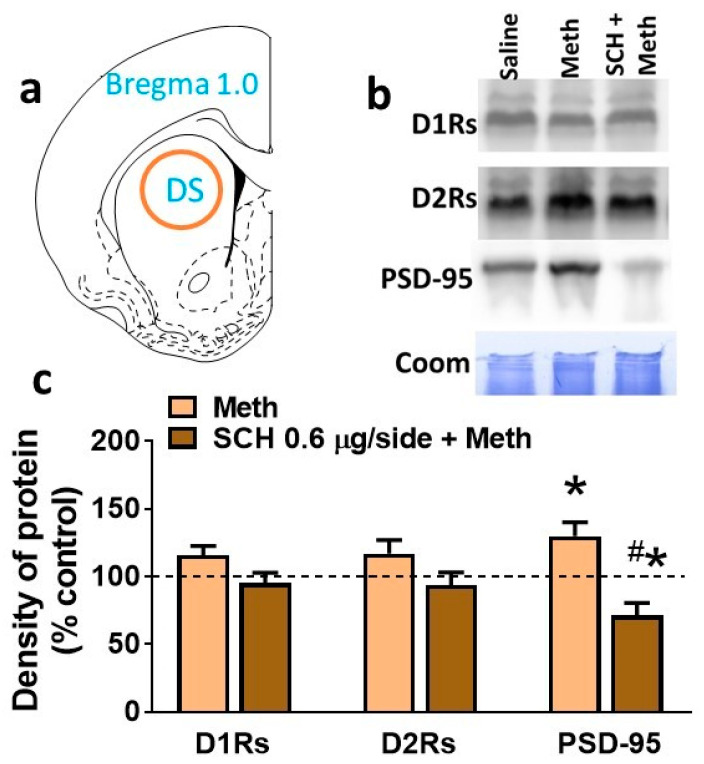
Methamphetamine self-administration increases the expression of PSD-95 in the dorsal striatum. (**a**) Schematic of a coronal slice representing the area of tissue punches collected for Western blotting analysis. (**b**) Representative immunoblots of the various proteins used for Western blotting analysis from control, methamphetamine, and SCH23390 + methamphetamine rats. Corresponding Coomassie staining (Coom) of the membrane is shown as loading control. (**c**) Quantitative analysis of protein expression in tissue from methamphetamine-exposed rats represented as a percentage change from saline controls. *n* = 5 saline self-administering rats without SCH23390/i.c. infusions, *n* = 4 saline self-administering rats with SCH23390, *n* = 9 methamphetamine self-administering rats without SCH23390/i.c. infusions, and *n* = 7 methamphetamine self-administering rats with SCH23390 infusion. Data are expressed as mean ± SEM. * *p* < 0.05 vs. controls; ^#^ *p* < 0.05 vs. methamphetamine rats by one-way ANOVA followed by post hoc analysis.

Expression of D_1_Rs, D_2_Rs, and PSD-95 was determined in dorsal striatal tissue homogenates in rats that self-administered methamphetamine and did not receive any infusions of SCH23390 or received SCH23390, and then compared with the expression of proteins in striatal tissue from rats that self-administered saline. One-way ANOVA did not detect a significant difference in D_1_Rs and D_2_Rs between the experimental groups. One-way ANOVA detected a significant difference in the expression of PSD-95 (F (2, 22) = 10.31, *p* = 0.007). Post hoc analysis revealed a higher expression of PSD-95 in methamphetamine rats, and this effect was inhibited and reduced in methamphetamine rats microinfused with SCH23390 (Figure 4c).

### 2.4. Basal Synaptic Transmission Is Compromised in Methamphetamine-Treated Slices, but Restored in the Presence of SCH23390

**Figure 5:**
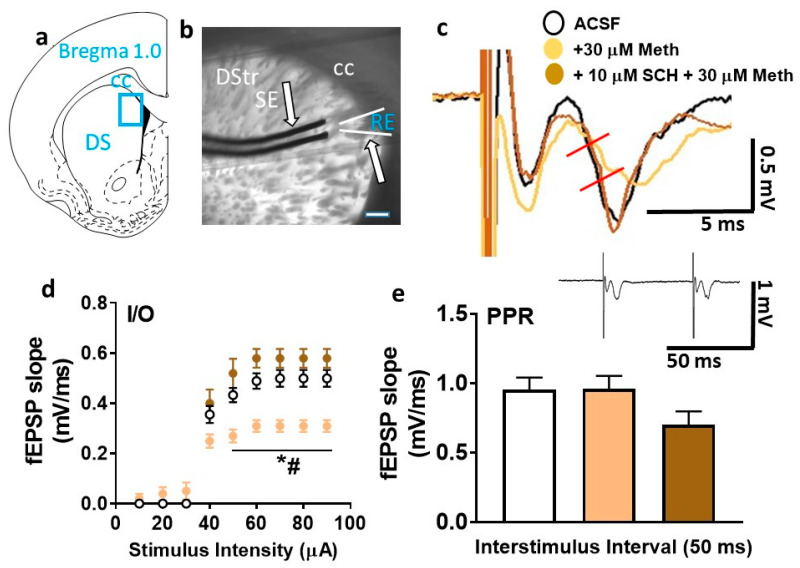
Basal synaptic transmission is reduced by superfusion of methamphetamine in the dorsal striatum. (**a**) Schematic of a coronal slice representing the area of the dorsomedial striatum in blue rectangular box used for stimulation and recordings. (**b**) Infrared microphotograph of a 440-µm-thick corticostriatal slice from one adult male rat indicating the location of the stimulating electrode (SE) and recording electrode (RE). The scale bar in (**b**) is 100 µm. Thick arrows point to the electrodes. CC: corpus callosum; DStr: dorsal striatum. (**c**) A representative field excitatory postsynaptic potential (fEPSP) waveform illustrating the parameter computed in the study, including fEPSP slope (measured between the two red lines) from control- (black trace), methamphetamine- (beige trace), and SCH23390 + methamphetamine-treated (brown trace) slices. (**d**) Input/output (I/O) curve obtained by plotting the slope of fEPSPs as a function of the stimulation intensity (from 10 to 90 µA) in the dorsomedial striatum. (**e**) Paired-pulse ratio (PPR) from each slice is averaged from at least six traces with interstimulus interval at 50 ms. Inset shows an example trace used to calculate PPR. Data are represented as mean ± SEM. *n* = 9 control slices, *n* = 10 methamphetamine slices, and *n* = 5 SCH23390 + methamphetamine slices. * *p* < 0.05 vs. controls; ^#^ *p* < 0.05 vs. SCH23390 + methamphetamine by Fisher’s least significant difference (LSD) post hoc test.

First, basal synaptic transmission was evaluated under control (artificial cerebrospinal fluid (ACSF)) and methamphetamine ± SCH23390 conditions. Repeated-measures two-way ANOVA with stimulus intensity and superfusion of methamphetamine ± SCH23390 (treatment) as independent variables and population spike slope as a dependent variable detected a significant treatment × stimulus intensity interaction (F (16, 168) = 14.2, *p* < 0.0001), a main effect of stimulus intensity (F (8, 168) = 314.8, *p* < 0.0001), and a main effect of treatment, (F (2, 21) = 12.6, *p* = 0.0002; Figure 5d). Post hoc analysis revealed that the input/output (I/O) curve was dramatically reduced in methamphetamine-treated slices compared to control- and SCH23390-treated slices, indicating that the excitability was reduced in methamphetamine-treated slices and restored in the presence of SCH23390.

### 2.5. Paired-Pulse Ratio Is Not Different among Treatment Groups

Short-term synaptic plasticity was assessed by measuring paired-pulse ratios. Paired-pulse ratio (PPR) was calculated as the change in slope of the second field excitatory postsynaptic potential (fEPSP) relative to that of the first fEPSP after an interstimulus interval of 50 ms. One-way ANOVA did not detect any treatment effects (F (2, 21) = 1.811, *p* = 0.18).

### 2.6. Synaptic Plasticity Is Reduced in the Presence of Methamphetamine, but Restored in the Presence of the D_1_R Antagonist SCH23390

**Figure 6:**
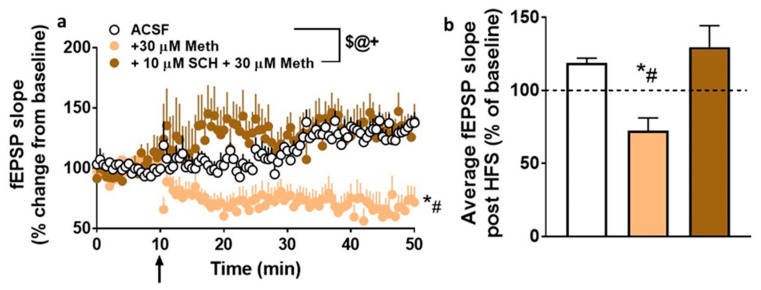
Ex vivo methamphetamine exposure reduces long-term potentiation (LTP) in dorsomedial striatal synapses. (**a**) *xy* graph of time course of fEPSPs before and after high-frequency stimulation (HFS) in all groups. The arrow in (**a**) at 10 min points to the time of HFS. (**b**) Average fEPSP slope of each experimental group post HFS (10 to 50 min). Data are represented as mean ± SEM. *n* = 8 control slices, *n* = 8 methamphetamine slices, *n* = 5 SCH23390 + methamphetamine slices. ^$^ *p* < 0.05 significant interaction; ^@^ *p* < 0.05 main effect of time; ^+^ *p* < 0.05 main effect of treatment by repeated-measures two-way ANOVA; * *p* < 0.05 vs. controls; ^#^ *p* < 0.05 vs. SCH23390 + methamphetamine by Fisher’s LSD post hoc test.

Under both control and methamphetamine + SCH23390 conditions, evoked fEPSPs showed significant LTP post HFS (Figure 6a). Two-way ANOVA with superfusion of methamphetamine ± SCH23390 (treatment) and time after HFS as independent variables and fEPSP slope as a dependent variable detected a significant treatment × time interaction (F (220, 1980) = 3.1, *p* < 0.0001), a main effect of treatment (F (2, 18) = 12.7, *p* = 0.0004), and a main effect of time (F (110, 1980) = 1.5, *p* = 0.006; Figure 6a). Post hoc analysis revealed significantly higher fEPSP slope in control (ACSF) and methamphetamine + SCH23390 conditions compared with the methamphetamine-treated condition.

We also measured the degree of LTP for each experimental group, visualized as the average fEPSP slope for the 40 min post-HFS recording period (Figure 6b). One-way ANOVA detected a significant effect of treatment (F (2, 18) = 12.6, *p* = 0.0004). Post hoc analysis revealed a significantly higher average fEPSP slope in control and methamphetamine + SCH23390 conditions compared with the methamphetamine only condition. Thus, these findings demonstrate that methamphetamine reduced synaptic plasticity in the dorsal striatum and SCH23390 prevented this effect.

## 3. Discussion

Our study used a pharmacological approach and evaluated the role of D_1_Rs in the dorsal striatum in altering the reinforcing properties of methamphetamine. We chose the dorsal striatum, as this neuroanatomical region has a rich dopaminergic innervation, regulates the transition from controlled to compulsive drug intake, and plays a role in stimulus-response habits [16,17,18]. Our findings demonstrate that site-specific antagonism of D_1_Rs in the dorsal striatum with SCH23390 reduces methamphetamine self-administration without effecting saline self-administration. In addition to the behavioral data, immunoblotting measures indicate that escalation of methamphetamine self-administration is associated with enhanced expression of PSD-95 in the dorsal striatum. Reduced self-administration after striatal microinfusion of SCH23390 is associated with decreased expression of PSD-95 in the dorsal striatum. Our site-specific pharmacological study highlights the locus of the protective effects of the antagonist on methamphetamine addiction-like behavior and can be indicative of an attenuation of the reinforcing properties of methamphetamine [24,25,26,27,28]. Furthermore, in striatal slices, electrophysiological findings demonstrate that, in the presence of methamphetamine, LTP is reduced and LTD is observed following HFS. More notable is that methamphetamine-induced LTD is prevented by SCH23390, indicating that D_1_Rs played a role in methamphetamine-induced synaptic depression. Taken together, these findings suggest that pharmacological inhibition of D_1_Rs in the dorsal striatum reduces methamphetamine intake and rescues methamphetamine-induced depression of synaptic plasticity.

These results suggest a role for dopaminergic mechanisms in the dorsal striatum in methamphetamine self-administration. Use of the fixed-ratio extended-access schedule of reinforcement in the current study enabled a comparison of effects of SCH23390 with effects previously obtained with a fixed-ratio limited-access schedule of reinforcement [24]. While both the studies confirm the mechanistic involvement of D_1_Rs in methamphetamine self-administration, our study provides additional support for dorsal striatal D_1_Rs in the reinforcing effects of the drug in animals that demonstrated escalation of methamphetamine intake. SCH23390 reduced responses during timeout in rats that self-administered methamphetamine, indicating that D_1_R antagonism also reduced responding when reinforcer was not available. However, it is unlikely that the decrease in methamphetamine self-administration is caused by an impaired ability to respond, as SCH23390 did not produce any behavioral effects in saline self-administering animals. Nevertheless, it is important to note that the current study examined the effects of dorsal striatal SCH23390 on response maintained under a simple fixed-ratio schedule of reinforcement. Therefore, a limitation in the interpretation of our findings is that they are restricted to drug intake and do not address the role dorsal striatal D_1_Rs play in reinforcing efficacy or motivation to self-administer methamphetamine. Additional studies with progressive-ratio schedules are required to test the role of D_1_Rs in the direction of reinforcement value or reinforcing power of methamphetamine [42,43].

We also determined neuroadaptations in the dorsal striatum that could play a role in reduced methamphetamine intake after microinfusion of SCH23390. For example, contingent methamphetamine via self-administration and non-contingent binge methamphetamine administration increases D_1_R binding and/or levels of D_1_Rs in the dorsal striatum in animal models and human subjects [44,45,46]. Changes in D_1_R binding and expression may be associated with altered expression of synaptic scaffolding proteins at the postsynaptic density in the dorsal striatum, as D_1_Rs are localized in the postsynaptic density [47]. Specifically, D_1_Rs interact with PSD-95, a prototypical scaffolding protein highly enriched in postsynaptic densities [47,48]. Notably, in vitro studies show that PSD-95 regulates agonist-mediated internalization of D_1_Rs [48]. Therefore, enhanced expression of PSD-95 in the dorsal striatum in methamphetamine-addicted rats may alter the D_1_R responsiveness to methamphetamine-stimulated dopamine levels and may regulate the reinforcing effects of the drug [49]. Furthermore, the reduction in the expression of PSD-95 in SCH23390-treated rats could be associated with normalization/inhibition of D_1_R responsiveness, including synaptic plasticity in the dorsal striatum together with reduced self-administration of methamphetamine [50,51].

In the dorsal striatum, electrophysiological studies demonstrate that repetitive activation of corticostriatal glutamatergic fibers produce LTP or LTD of excitatory synaptic transmission [52,53]. For example, LTP and LTD induction in corticostriatal synapses occur via activation of D_1_Rs [53,54,55]. With respect to dopamine and psychostimulants, ex vivo studies in the ventral striatum demonstrate that superfusion of dopamine, cocaine, or amphetamine reduces excitatory synaptic transmission via D_1_Rs [56]. We, therefore, examined and characterized the acute effects of methamphetamine on basal synaptic transmission, measured as I/O curves, and synaptic plasticity, measured as HFS-induced LTP in dorsal striatal slices. We then investigated whether methamphetamine-induced effects on synaptic transmission and synaptic plasticity were mediated by D_1_Rs. We demonstrate that superfusion of methamphetamine reduced baseline synaptic transmission, indicating that acute methamphetamine reduces basal excitatory drive in the dorsomedial synapses. Bath application of SCH23390 completely blocked the effect of methamphetamine on basal synaptic transmission. We also measured paired-pulse ratio in dorsomedial striatal synapses, as this provides a measure of short-term plasticity. For example, a change in paired-pulse ratio supports either facilitation or depression of synaptic strength, and it is thought to reflect activity-dependent changes of transmitter release from presynaptic terminals [57]. We report that paired-pulse ratio did not differ in the dorsomedial striatal synapses under superfusion of methamphetamine and was not altered by SCH23390. Therefore, our results highlight the fact that acute methamphetamine spares presynaptic sites in the dorsomedial striatal synapses, and they suggest that methamphetamine-mediated reduction of basal synaptic transmission probably occurs through postsynaptic events. Our findings also show that methamphetamine altered HFS-induced LTP in the dorsomedial striatum. In fact, superfusion of methamphetamine produced LTD after HFS. Furthermore, bath application of SCH23390 completely blocked the effect of methamphetamine on synaptic plasticity. These novel findings suggest that the effects of methamphetamine on synaptic transmission and plasticity are mediated via activation of D_1_Rs in the dorsal striatum.

The acute effects of methamphetamine on HFS-induced LTP reported here support a recent study which demonstrated that superfusion of methamphetamine inhibits HFS-induced LTP in the hippocampus [58]. Even more significant is that HFS-induced reduction in LTP in the hippocampus is blocked by SCH23390 and is not affected by the D_2_R antagonist (eticlopride) or the NMDA receptor antagonist (dl-2-amino-5-phosphonovaleric acid) [58]. Combining these findings with our current data, they indicate that acute methamphetamine-induced synaptic plasticity in the dorsal striatum and hippocampus is D_1_R-dependent. As a mechanistic hypothesis, we speculate that the indirect activation of D_1_Rs by methamphetamine in the dorsal striatum may be altering the endocannabinoid system to attenuate corticostriatal glutamatergic input to induce LTD [33]. Taken together, it appears that aberrant D_1_R function in the dorsal striatum by methamphetamine produces synaptic depression in the dorsal striatum, and inhibition of D_1_R function may correlate with normalizing synaptic plasticity in the dorsal striatum and reducing the reinforcing properties of methamphetamine. Such studies are an important future pursuit.

## 4. Material and Methods

### 4.1. Animals

All animal procedures were approved by the Institutional Animal Care and Use Committee (IACUC) of VA San Diego Healthcare System (protocol #A16-000, approved 29-04-2016). Forty-one adult male Long–Evans rats (weighing 300–350 g at the start of the experiment, bred at the VA Vivarium) were housed two per cage in a temperature-controlled vivarium under a reverse light/dark cycle (lights off 9:00 a.m.–9:00 p.m.) and completed the study.

### 4.2. Intravenous Catheterization Surgery

Twenty-nine rats underwent surgery for catheter implantation for intravenous self-administration. Intravenous surgery, catheter maintenance, and catheter patency were performed according to our previous publications [59,60].

### 4.3. Intracranial Cannula Surgery

Immediately following intravenous catheterization surgery, 11 rats were prepared for cannulation surgery. Indwelling guide cannulae (26 gauge, Plastics One) were bilaterally placed under anesthesia (2–3% of isofluorane/oxygen mixture) in a stereotaxic instrument (David Kopf Instruments). Guide cannulae were positioned 1 mm above the desired area in the dorsal striatum and anchored to the skull with four stainless-steel screws and dental acrylic cement. The coordinates (in mm, relative to bregma) used for placement of intracranial cannulae were as follows: anterior posterior (AP), 1.0 mm from bregma; medial lateral (ML), ±2.3 mm from bregma; dorsal ventral (DV), −4.0 from dura. Immediately after surgery, Flunixin^®^ (2.5 mg/kg, s.c.) was given as an analgesic, and Cefazolin was administered as an antibiotic.

### 4.4. Intravenous Methamphetamine Self-Administration

*Training and maintenance on the extended-access schedule (days 1–6):* Following four days of recovery after surgery, 20 rats (thirteen with i.v. cannula, seven with i.c. and i.v. cannula) were trained to press a lever according to the fixed-ratio 1 (FR1) schedule of methamphetamine reinforcement (0.05 mg/kg/injection of NIDA-provided methamphetamine) in operant boxes (Med Associates) under extended-access conditions (6 h access per day for six days). A response on the active lever resulted in a 4 s infusion (90–100 μL of methamphetamine), followed by a 20 s timeout period to prevent overdose. Each infusion was paired for 4 s with white stimulus light over the active lever. Responses during the timeout or on the inactive lever were recorded but resulted in no programmed consequences. All animals increased methamphetamine self-administration. FR data were analyzed as number of reinforced or non-reinforced (i.e., during the timeout period) lever presses per session.

### 4.5. Intravenous Saline Self-Administration

*Training and maintenance on the extended access schedule (days 1–6):* Following four days of recovery after surgery, nine rats (five with i.v. cannula, 4 with i.c. and i.v. cannula) were trained to press a lever for i.v. saline (0.9% sterile) in an operant chamber similar to the paradigm used for methamphetamine self-administration (FR1; 6 h per session).

### 4.6. Intracranial Saline and SCH23390 Infusions

Intracranial infusions were carried out in saline- and methamphetamine-treated rats when stable performance was established (days 7–9). Initially, during a sham infusion session, animals were habituated to insertion of the injectors into the guide cannulae (33 gauges extending 1 (±0.04) mm beyond the guide cannulae) (Plastics One, model C235). During the infusion experiments, saline or SCH23390 was infused 5 min prior to the self-administration session during which no i.v. infusions were conducted. Rats were bilaterally infused with either saline or SCH23390 over a period of 1 min at a rate of 0.3 μL/min using 10 μL Hamilton syringes driven by a syringe infusion pump (Harvard Apparatus). Following infusion, the injectors remained in place for an additional 30 s to allow diffusion of saline or SCH23390. The order of testing was saline followed by SCH23390 (0.3, 0.6 µg/side in 0.3 µL volume [43,61]). Each rat received only one infusion of saline and each dose of SCH23390. Based on previous reports, a similar injection volume of SCH23390 spread about 3 mm^3^ into the adult rat brain 120 min after microinfusions [43]. Therefore, we believe that SCH23390 stayed within the targeted dorsal striatal area. However, a limitation with such procedures is the fact that the entire dorsal striatal area may not be covered with such volume/flow rate and, therefore, in our case, all D_1_Rs in the extended dorsal striatal area were not targeted. Additionally, SCH23390 has affinity to serotonergic 5-HT2 receptors, albeit at higher doses [62].

### 4.7. Brain Tissue Collection for Immunohistochemistry and Western Blotting

Saline- and methamphetamine-treated rats were euthanized by rapid decapitation under light isoflurane anesthesia (3–5%) 1 h after conclusion of the FR session. The left hemisphere was snap-frozen for Western blotting and the right hemisphere was post-fixed in 4% paraformaldehyde for immunohistochemistry.

### 4.8. Determination of Cannula Placement

Right hemisphere of the brain tissue containing the track of cannula (Figure 3b) was sliced in 40 μm sections along the coronal plane in a cryostat. Brain tissue was visualized for cannula placement. All brains showed placement in the correct area in the dorsal striatum (Figure 3c).

### 4.9. Western Blotting

Tissue punches from 300-μm-thick sections of dorsal striatum from saline (*n* = 5 without i.c. infusions; *n* = 4 with i.c. infusions) and methamphetamine-treated rats with (*n* = 7) or without (*n* = 9) i.c. cannulas encompassing the injection site were homogenized on ice by sonication in MES buffer (150 mM NaCl, 2 mM EDTA, and 150 mM Na_2_CO_3_, 1 mM EDTA with Protease Inhibitor Cocktail and Phosphatase Inhibitor Cocktails II and III diluted 1:100), heated at 70 °C for 10 min, and stored at 80 °C until determination of protein concentration by a detergent-compatible Lowry method (Bio-Rad). Samples were mixed (1:1) with a Laemmli sample buffer containing β-mercaptoethanol. Protein samples (20 μg) were run on 10% SDS-PAGE gels (Bio-Rad, Hercules, CA, USA) and transferred to polyvinylidene fluoride membranes (PVDF pore size 0.2 μm). Membranes were blocked with 5% milk (*w*/*v*) in TBST (25 mM Tris–HCl, pH 7.4, 150 mM NaCl and 0.1% Tween 20 (*v*/*v*)) for 2–4 h at room temperature and were incubated with the primary antibody for 16–20 h at 4 °C: antibody for D_1_R (1:1000, AB20066 Abcam, Cambridge UK), dopamine D_2_ receptors (D_2_R, 1:500, AB5084P Sigma Aldrich, St.Louis, MO, USA), and PSD-95 (1:1000, MA1-045 Invitrogen, Waltham, MA, USA). Membranes were then washed with TBST and incubated for 1 h at room temperature with horseradish peroxide-conjugated goat antibody to rabbit (1:1000 for D_1_R, D_2_R) or horseradish peroxide-conjugated goat antibody to mouse (1:1000 for PSD-95) in TBST. Following subsequent washes, immunoreactivity was detected using SuperSignalWest Dura chemiluminescence detection reagent (Thermo Scientific, Waltham, MA, USA) and images were collected using a digital imaging system (Azure Imager c600). For normalization purposes, membranes were incubated with 0.125% Coomassie stain for 5 min and washed three times for 5–10 min in destaining solution. Densitometry was performed using ImageJ software (NIH). The signal value of the band of interest following subtraction of the background calculation was then expressed as a ratio of the corresponding Coomassie signal (following background subtraction). This ratio of expression for each band was then expressed as a percentage of the saline rat included on the same membrane.

### 4.10. Slice Preparation for Electrophysiology

Age-matched male rats (*n* = 12) were anesthetized with isoflurane and killed by rapid decapitation. Brains were quickly removed and placed in ice-cold artificial cerebrospinal fluid (ACSF) containing (in mM) 125 NaCl, 26 NaHCO_3_, 4 KCl, 1.25 NaHPO_4_, 2 CaCl_2_, 1 MgCl_2_, and 10 glucose bubbled with 95% oxygen and 5% CO_2_ [63]. Brains were trimmed on the dorsal side at an angle of approximately 140° from the horizontal plane and glued to a vibratome base (Leica VT1000S). Thick slices (440 μm) containing cortico-striatal projections were obtained and used for recordings. Two to three slices (representing 1.6 to 1.0 mm from bregma) per rat were transferred to a submerged chamber and incubated with oxygenated ACSF at room temperature for at least 1.5–2 h before initiating recordings. Recordings were made in the dorsomedial striatum (Figure 5b) in a submersion-type recording chamber superfused with oxygenated ACSF at a rate of 2–3 mL/min at room temperature and positioned on the stage of an upright motorized microscope (Olympus BX51 WI, Scientifica) equipped with a back Illuminated sCMOS camera (Prime 95B, Photometrics) and a broad-spectrum light-emitting diode (LED) illuminator (pE-300, CoolLED).

### 4.11. Field Potential Recordings

To study basal synaptic transmission, paired-pulse ratio, and synaptic plasticity under control, methamphetamine (30 µM) [58], and SCH23390 (10 µM) [64] plus methamphetamine conditions, local field potentials were recorded in acute brain slices. Population spikes or field excitatory postsynaptic potentials (fEPSPs) were evoked by extracellular stimulation (0.03 Hz, 0.2 ms) in the dorsomedial striatum using a silver-coated tungsten wire stimulating electrode (50 μm, A-M System; Figure 5b). fEPSPs were recorded using ACSF-filled patch pipettes with tip resistances of 2–4 MΩ. Pipettes were pulled from borosilicate glass capillaries (PG150T-10, Harvard Apparatus) using a micropipette puller (PC-10, Narishige). At least two slices per rat were used for recordings. Slices were recorded under control (ACSF) conditions (*n* = 9), after superfusion of 30 µM methamphetamine in ACSF (*n* = 10), or after superfusion of 10 µM SCH23390 + 30 µM methamphetamine (*n* = 5). Slices were continuously super-perfused with drugs for at least 20 min before the start of each recording session and until the end of the experiment.

Basal synaptic transmission was analyzed by generating stimulus/response curves or input/output (I/O) curves prior to each synaptic plasticity experiment. I/O curves were generated by plotting stimulus intensity (10–90 µA) versus fEPSP slope (Figure 5d) [65]. The slopes of fEPSPs were measured after the stimulus artifact and the fiber volley from the initial 2 to 5 ms of the rising phase to about half-peak time of the synaptic response. For the remainder of the experiment, the test stimulus intensity was set to elicit a fEPSP that is approximately 40–50% of the maximum response recorded during the I/O measurements. Paired-pulse ratios (P2/P1) were evaluated by dividing fEPSP of the second slope by the first slope obtained after a 50 ms inter-stimulus interval (Figure 5e). fEPSPs at this constant test stimulus intensity were monitored for a period of 25 min to ensure a stable response before induction of LTP.

For induction of synaptic plasticity or LTP in dorsal striatal synapses, the following HFS stimulation paradigm was used: four 1 s, 100 Hz trains delivered 10 s apart [66]. For comparisons of treatment effects on fEPSP slope between slices, values for each recording were normalized to the average slope for the 10 min of baseline before HFS was initiated. Data were acquired, filtered (high pass, 0.1 Hz; low pass, 3 kHz), and amplified a using a computer-controlled patch-clamp amplifier (MultiClamp 700B, Molecular Devices, San Jose, CA, USA) and digitized using an analog-to-digital converter (Digidata 1550A1, Molecular Devices). Analysis of fEPSP slopes was performed using pClamp10.4 software (Molecular Devices).

### 4.12. Statistical Analyses

The methamphetamine and saline self-administration data are expressed as the average total number of lever responses per session. Methamphetamine or saline self-administration during the 6 h FR sessions was examined using a two-way repeated-measures analysis of variance (ANOVA) followed by the uncorrected Fisher’s least significant difference (LSD) post hoc test. Active, inactive, and timeout lever presses were analyzed separately. A one-way ANOVA was conducted to determine differences between saline, methamphetamine only, and methamphetamine + SCH23390 groups for Western blots of all proteins followed by Tukey’s post hoc tests for each protein. Western blot analysis was conducted on percentage change values from saline controls. Data are expressed as mean ± standard error of the mean (SEM) in all graphs. Effects of methamphetamine and methamphetamine + SCH23390 on fEPSP slope were analyzed using a two-way ANOVA. Effects on paired-pulse ratio were analyzed using one-way ANOVA. Post hoc analyses were conducted when a significant interaction was detected. Significance was set at *p* < 0.05. SPSS version 19 (IBM, Armonk, NY, USA) or GraphPad Prism version 7 (GraphPad, La Jolla, CA, USA) were used for statistical analysis.
